# Aspergillosis of the Heart and Lung and Review of Published Reports on Fungal Endocarditis

**DOI:** 10.1007/s11046-016-0012-9

**Published:** 2016-05-31

**Authors:** Beata Sulik-Tyszka, Piotr Kacprzyk, Krzysztof Mądry, Bogna Ziarkiewicz-Wróblewska, Wiesław Jędrzejczak, Marta Wróblewska

**Affiliations:** Department of Microbiology, Public Central Teaching Hospital in Warsaw, Warsaw, Poland; Department of Haematology, Oncology and Internal Medicine, Medical University of Warsaw, Warsaw, Poland; Department of Pathomorphology, Medical University of Warsaw, Warsaw, Poland; Department of Dental Microbiology, Medical University of Warsaw, 1a Banacha Street, 02-097 Warsaw, Poland

**Keywords:** Invasive aspergillosis, Heart, Lung, Stem cell transplantation, Amphotericin B

## Abstract

Invasive aspergillosis (IA) is increasingly diagnosed in high-risk patients. The lesions are usually located in the lungs and/or sinuses, and the fungus may spread haematogenously to different organs; however, involvement of the heart during IA is very rare. We describe a unique case of invasive aspergillosis of the heart septum and the lungs in the allogeneic haematopoietic stem cell transplant recipient.

## Introduction

Patients receiving chemo- and/or radiotherapy treated with glucocorticosteroids and immunosuppressive agents are at high risk of systemic fungal infection [1, 2]. Another high-risk group constitutes patients with acquired immune deficiency syndrome (AIDS), solid organ transplant (SOT) recipients and with congenital immune deficiencies [1]. At particularly high risk of systemic fungal infections, compared to other groups of patients, are patients hospitalised in haematological wards [3]. Risk factors for these infections are neutropaenia, particularly deep (<500 neutrophils/mm^3^) and long lasting (>7 days), and lymphopaenia, pertaining especially to CD4+ cells.

However, the highest risk of systemic fungal infections is reported in the recipients of allogeneic haematopoietic stem cell transplants [2]. Furthermore, graft-versus-host disease (GVHD) and its treatment with corticosteroids make these patients susceptible to fungal infections caused by moulds classified in the genus *Aspergillus* or yeast-like fungi of *Candida* spp. [4]. Moreover, in the aspect of defective immune response, the chronic form of GVHD causes functional asplenia in these patients.

The most common aetiological factors of invasive fungal infection (IFI) remain *Candidaalbicans* and *Aspergillus fumigatus* [3]. The broad use of fluconazole in prophylaxis of these infections caused an increase in isolation rates of *Candida* non-*albicans* strains as well as a further increase in frequency of *Aspergillus* spp. infections. Among patients with invasive aspergillosis with pneumonia and sinusitis, with subsequent haematogenous spread to other organs, mortality may reach 80–90 % [5–10].

## Case Report

A 45-year-old man was admitted as an emergency to the haematology ward because of fever, nausea, vomiting, diarrhoea and severe prostration, which have been lasting for several days. The patient suffered from a mantle cell lymphoma and was in the 45th day after allogeneic transplantation of haematopoietic stem cells (alloHSCT) from a related donor, after myeloablative conditioning. On physical examination tachycardia, hypotension, high levels of inflammatory markers and marked dehydration were detected. Laboratory tests showed acute renal insufficiency of complex aetiology: prerenal (due to dehydration) and intrinsic—with the symptoms of thrombotic thrombocytopenic purpura (TTP) and kidney damage, which resulted from cyclosporine nephrotoxicity. In histopathological examination of biopsy specimens obtained during sigmoidoscopy, acute graft-versus-host disease (aGVHD) had been diagnosed. During high-resolution computed tomography (HR-CT) scan of the chest, a typical radiological picture of pulmonary mycosis was detected. Galactomannan (GM) has been detected in the patient’s serum, while the test for the presence of GM in the bronchoalveolar lavage (BAL) fluid was not done. Therapy with lipid complex amphotericin B was administered (5 mg/kg/day), and control examinations showed a slow regression of inflammatory lesions in the lungs. Due to a marked fur on oral mucous membranes, several mycological cultures were done, which yielded the growth of *Pichia kluyveri* and *Candida albicans*. Mycological cultures of BAL and sputum were negative. Treatment with lipid complex amphotericin B was continued.

During subsequent weeks of hospitalisation, the patient was treated for hypogammaglobulinaemia, reactivation of cytomegalovirus (CMV), Epstein–Barr virus (EBV) and adenovirus in peripheral blood, as well as for *Giardia lamblia* infection of the gastrointestinal tract. He was also diagnosed with papillary carcinoma of the thyroid gland, for which strumectomy was performed. Two episodes of ileus were recorded during hospital stay.

During the patient’s hospitalisation, several evaluations of post-transplant chimerism were done. Results of all tests (done on peripheral blood and on bone marrow samples taken >100 days after alloPBSCT) were normal. Myelogram of the trepanobiopsy specimen revealed dysmegakaryopoiesis; however, phenotyping of the bone marrow cells did not show lymphoma relapse. Cytogenetic tests also did not show aberrations. HR-CT scans of the chest, abdominal cavity and neck were performed in search for possible relapse of the basic disease. A single enlarged (dimension of 19 mm), probably reactive, lymph node was detected in the abdominal cavity.

Due to episodes of septic shock, the patient was treated with antibiotics (imipenem, colistin, linezolid, tigecycline), an antiviral agent (acyclovir) and a lipid complex of amphotericin B, as well as a granulocyte colony stimulating factor (G-CSF). At that stage, laboratory tests showed increasing levels of inflammatory markers as well as increasing lactic acidosis and its respiratory compensation. The patient required oxygen therapy.

Pulmonary embolism was excluded during bedside echocardiography examination. The lipid complex of amphotericin B was temporarily discontinued (after 93 days of therapy) in fear for development of acidosis as an unwanted effect of this antifungal agent (renal canalicular and tubular acidosis). Furthermore, clinical symptoms of acute pancreatitis and a sudden increase in pancreatic enzyme levels had been recorded. During subsequent days, the patient suffered from increasing shortness of breath; however, in a chest X-ray examination neither obvious parenchymal lung lesions nor haemostasis in the pulmonary circulation had been detected. After nephrological consultation of the patient, a dialysis catheter had been inserted into the femoral vein, and therefore, amphotericin B therapy was not continued. Due to an increasing metabolic acidosis (pH 7.3) and lactate levels, symptoms of acute pancreatitis and persistent paralytic ileus, a CT scan of the abdominal cavity was performed to exclude mesenteric artery thrombosis and intestinal ischaemia as a possible cause of these abnormalities. However, no mesenteric embolism or clear signs of acute pancreatitis were detected. There were also no indications for surgical intervention. The patient’s condition continued to deteriorate, and he was transferred to the intensive care unit (ICU). On admission to the ICU, his condition was severe, with borderline respiratory function. His status of consciousness was assessed as score 14 of the Glasgow Coma Scale (GCS). Haemodialysis was performed; however, despite intensive therapy, hypoglycaemia and acidosis were deepening and the patient died.

**Fig. 1:**
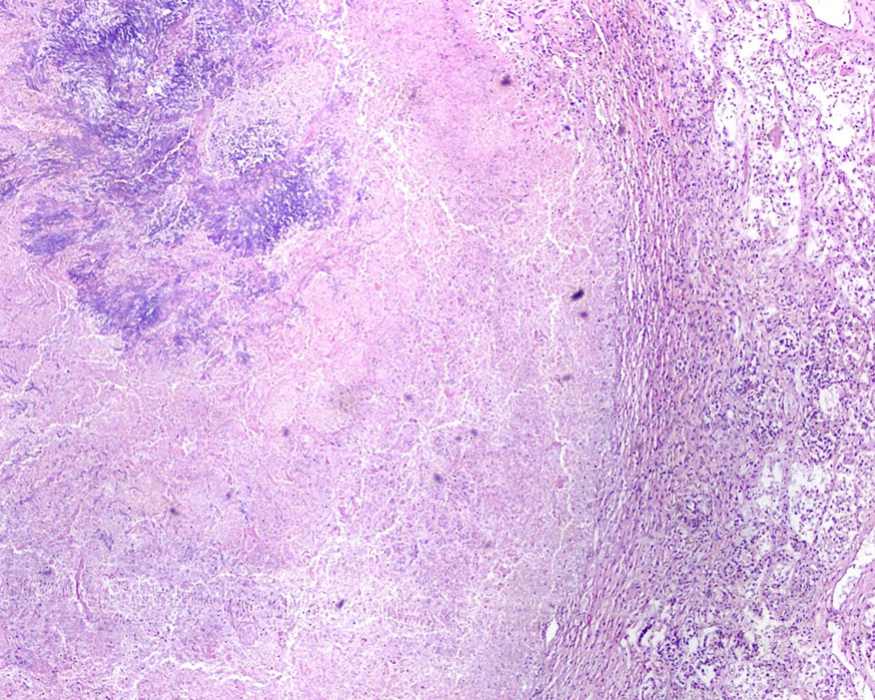
Lung: necrosis within the *Aspergillus* spp. mycelium. Haematoxylin and eosin staining. Magnification ×40

**Fig. 2:**
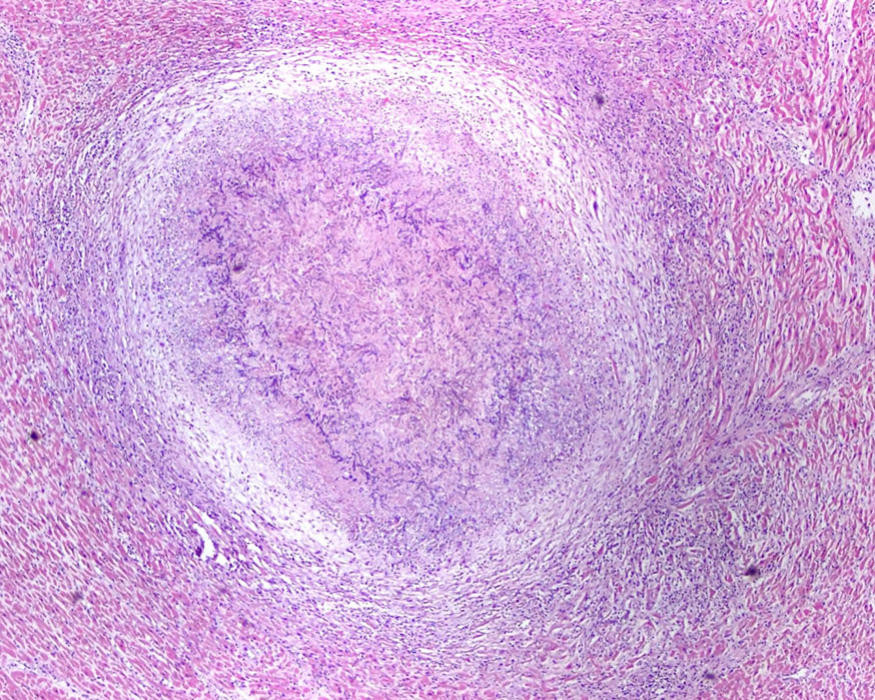
Heart: spherical mycelium of *Aspergillus* spp. with cardiomyocytes visible around it. Haematoxylin and eosin staining. Magnification ×40

**Fig. 3:**
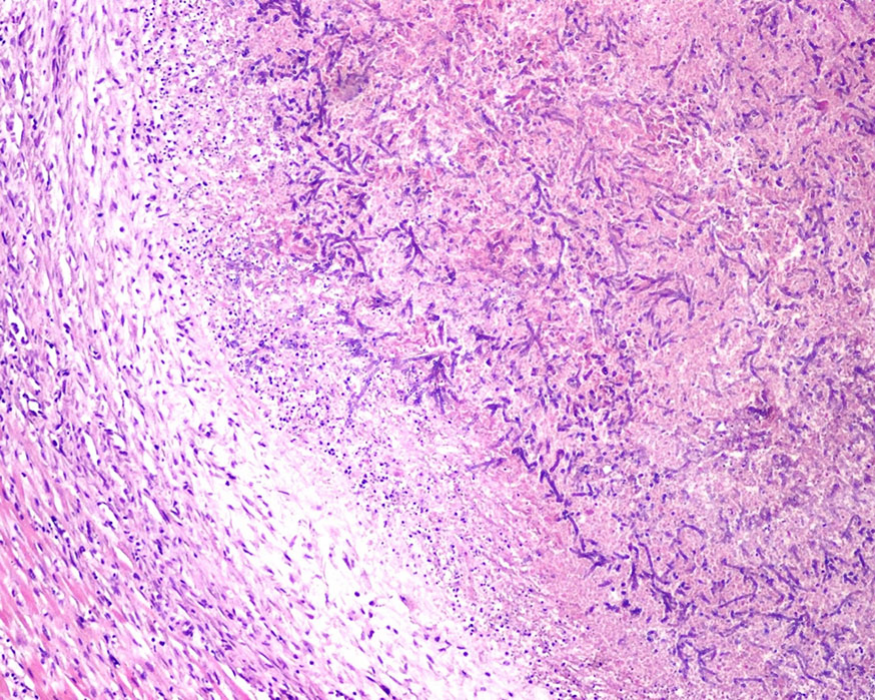
Heart: *Aspergillus* spp. mycelium with branching hyphae. Small mixed inflammatory infiltrations seen around it. Haematoxylin and eosin staining. Magnification ×100

**Fig. 4:**
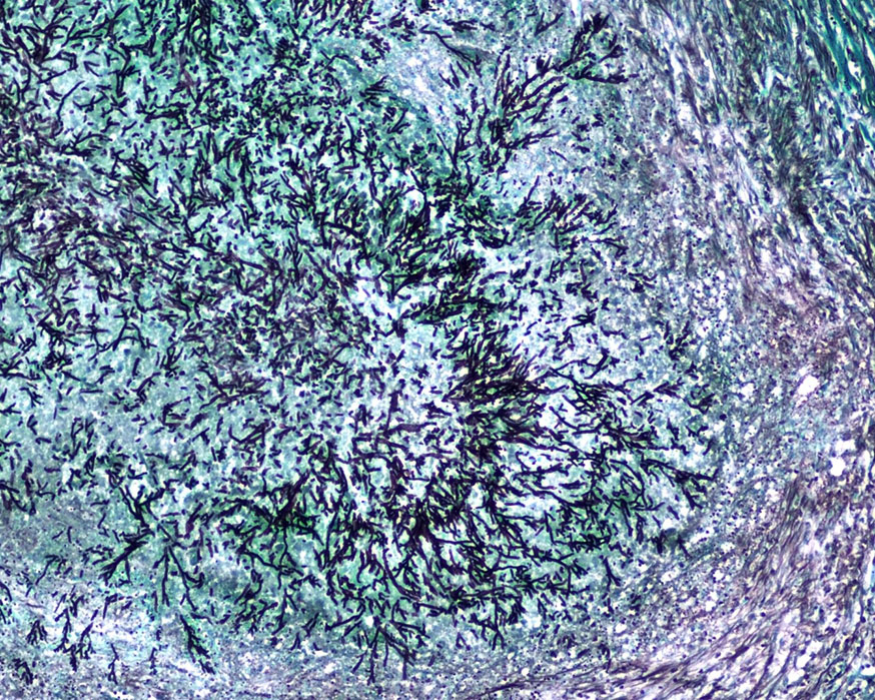
Heart: radial arrangement of *Aspergillus* spp. hyphae. Grocott’s methenamine silver staining. Magnification ×100

Blood culture of a sample obtained just before his death had shown the presence of non-fermenting Gram-negative rod *Brevundimonas nasdae*. No fungi were cultured. An autopsy examination revealed marked hyperaemia and tissue oedema in both lungs, as well as irregular regions of increased consistency, particularly in the lower lobes of both lungs. Microscopically, the lesions had been described as organising abscesses with necrosis in the central zone and the presence of hyphae typical for *Aspergillus* spp. (Fig. 1). In the interventricular septum of the heart, hypertrophy of single cardiomyocytes had been noted, as well as an abscess with *Aspergillus* spp. hyphae present within the central necrosis zone (Figs. 2, 3, 4).

## Discussion

In the described case, allogeneic transplantation of peripheral blood haematopoietic stem cells (alloPBHSCT) constituted a major risk factor for IFI caused by moulds classified in the genus *Aspergillus*. Fungal lesions with hyphae characteristic for *Aspergillus* spp. had been detected in anatomopathological investigations both in the heart and in the lungs, despite treatment of the patient with amphotericin B, according to the Infectious Diseases Society of America (IDSA) guidelines. Furthermore, after transplantation the patient suffered from GVHD. This complication develops in 60–80 % of alloHSCT recipients. It results from a reaction of allogeneic T lymphocytes, derived from the transplanted cells and directed against the recipient’s antigens.

A disease developing within 100 days after transplantation is called an acute GVHD. In the case of alloHSCT, recipients remain in deep immunosuppression, which makes them prone to IFIs, caused by yeast-like fungi of the genus *Candida*, as well as by moulds *Aspergillus* spp. [4]. The most common is invasive aspergillosis (3–14 %). The fungal spores of *Aspergillus* are inhaled; therefore, the most common clinical forms are infections of the respiratory tract and systemic infections, involving all types of organs, including the heart, although this location is very rare [11–14].

Invasive aspergillosis often begins with invasion of the blood vessels; their obstruction caused by thrombus with mycelium, as well as with haemorrhagic infarctions, which are very characteristic for this type of infections [15]. The infarction zone comprises ischaemic necrosis caused by blood vessel blockage. Hyphae of the mould are seen within the necrotic tissues. Haematogenous spread may involve the brain, heart, kidneys and gastrointestinal tract mucous membranes. The presence of *Aspergillus* hyphae in the tissue justifies the diagnosis of invasive aspergillosis; however, it is recommended to send such material also for mycological culture for species identification.

Bapat and Young [16] reported a case of fungal endocarditis in a 73-year-old man, in whom this complication followed surgical replacement of the aortic valve and coronary artery bypass surgery (Table 1). A diagnosis of fungal endocarditis on clinical grounds alone is very difficult because of non-specific symptoms, such as fever (72 %), embolic episodes (69 %), new or changing heart murmurs (41 %) or sudden visual loss (13 %) [17]. Diagnosis is further complicated by the fact that blood cultures are usually negative [2, 3]. Unfortunately, very often a diagnosis is made at post-mortem examination. In the described case, a laboratory diagnosis was made on the basis of histopathological tests of the thrombus removed during embolectomy, as endocarditis may rarely cause emboli in the peripheral blood vessels. To avoid a relapse of the fungal infection, it is recommended to perform surgery and administer long-term antifungal therapy with amphotericin B [16].

**Table 1:** Published reports on fungal endocarditis

Publication [ref. no.]	Clinical presentation	Laboratory diagnosis
Bapat and Young [16]	Fungal endocarditis in a 73-year-old man, after surgical replacement of the aortic valve and coronary artery bypass surgery	Histopathological examination of the thrombus removed during embolectomy
Alvarez et al. [18]	Fungal endocarditis in a 12-year-old girl with acute lymphoblastic leukaemia at haematological remission	Vegetations detected in echocardiography; culture of the vegetation removed during surgery (growth of *A. terreus*)
Abuzaid et al. [19]	Fungal endocarditis in a 64-year-old woman who 1 month earlier underwent mitral valve repair surgery	Severe mitral regurgitation observed in echocardiography; isolation of a strain of *A. fumigatus* from the heart valve removed during surgery
Satish et al. [20]	Extensive cardiothoracic aspergillosis in a 47-year-old man no risk factors for immunosuppression	Echocardiography, magnetic resonance imaging and computed tomography: extensive infiltration of the atria and ventricles by the mediastinal lesion; histopathological examination of the subcutaneous nodule on the chest revealed *Aspergillus* granuloma (growth of *A. fumigatus* in culture); fine-needle aspiration cytology from the mediastinum revealed *Aspergillus* granuloma; no fungal mycelium was detected in the endomyocardial biopsy from the right atrial side of the atrial septum

Alvarez et al. [18] described a case of a 12-year-old girl with acute lymphoblastic leukaemia at haematological remission, hospitalised due to fever, nausea, vomiting, abdominal pains and pancytopaenia. Upon admission, a combined antimicrobial therapy with vancomycin, gentamycin and amphotericin B was started. During echocardiography, masses had been noticed in the apex of the left ventricle of the heart. She underwent surgery, and culture of clinical specimens revealed the growth of *Aspergillus terreus*. Embolic infarctions of the spleen, liver and the kidneys were detected in CT. There were no symptoms or signs of relapse of endocarditis caused by *Aspergillus* spp.; however, 28 days after surgery she suffered from severe headaches and dizziness. CT scan showed cerebral haemorrhage, which comprised the right temporal lobe of the brain. The patient died the following day. Due to an increasing number of patients treated with immunosuppressive agents, as well as an increase in frequency of fungal infections caused by *Aspergillus* strains, early diagnosis and proper antifungal therapy increase the chance for patients’ survival. However, in the described case the patient died despite rapid surgical intervention. It is therefore important to stress that fungal endocarditis in patients with acute leukaemia may compromise their prognosis and decrease the rate of survival [18].

Abuzaid et al. [19] published a case of a 64-year-old woman admitted to an emergency ward due to acute respiratory failure, with several underlying diseases: diabetes, arterial hypertension and hypercholesterolaemia. Severe mitral regurgitation was observed in echocardiography, and the patient was qualified for surgical valve replacement. Vancomycin and gentamycin were administered as empiric therapy. Several days later, aspergillosis of the heart was confirmed by the isolation of a strain of *A. fumigatus* from her heart valve removed during surgery and treatment was changed to intravenous voriconazole. Both rapid surgical intervention and proper antifungal therapy improve prognosis of patients with infective endocarditis caused by *Aspergillus* spp. Similarly, early administration of proper antifungal therapy and fast surgical intervention also improve the chance for survival of patients with invasive aspergillosis of the heart. However, due to non-specific clinical symptoms (fever, episodes of thrombosis, heart murmurs) additional tests—such as histopathology, echocardiography, mycological cultures and serological assays—are useful [2].

Satish et al. [20] described a case of cardiothoracic aspergillosis, although no fungal mycelium was detected in the heart. We report a case of invasive aspergillosis located in the interventricular septum of the heart and in both lungs of an immunocompromised patient. On histopathological examination, hyphae typical for *Aspergillus* spp. were observed. The patient died despite therapy with amphotericin B, according to the IDSA guidelines. Recently it has been suggested that combination therapy with voriconazole and anidulafungin may improve the outcome of IA [21]. It is known that strains of *Aspergillus* spp. may form a biofilm, which might be disrupted by echinocandins, while azoles do not penetrate it.

Due to an increasing number of patients on immunosuppressive therapy and an increase in frequency of cases of invasive aspergillosis, in each case of probable infection of this aetiology, other tests (such as histopathology, imaging, non-culture diagnostic assays)—apart from clinical signs and symptoms—are recommended, as they may help with earlier diagnosis and improved survival of these patients [3, 19, 22].
